# Disseminated Tuberculosis Masquerading as Malignancy in an Immunocompetent Middle-aged Woman: A Multiorgan Imaging Case Report and Updated Review for Clinicians

**DOI:** 10.2174/0115734056432546251217062302

**Published:** 2026-01-13

**Authors:** Jacobo-Enrique Adam-Sosa, Andrea-Fernanda Gonzalez-Soto, Luis Camarillo-Solache, Ricardo Cebrian-Garcia, Mauricio Molina-Gonzalez, Maria-del-Carmen Garcia-Blanco, Ernesto Roldan-Valadez

**Affiliations:** 1 Radiology Department, Hospital Angeles Acoxpa, 14308, CDMX, Mexico;; 2 Department of Medical Education, Hospital Angeles Acoxpa, 14308, CDMX, Mexico;; 3 Orthopedic Unit. 14308, Hospital Angeles Acoxpa, CDMX, Mexico;; 4 Intensive Care Unit. 14308, Hospital Angeles Acoxpa, CDMX, Mexico;; 5 Division of Research, Instituto Nacional de Rehabilitacion 'Luis Guillermo Ibarra Ibarra', 14389, Mexico City, Mexico;; 6 I.M. Sechenov First Moscow State Medical University (Sechenov University), Department of Radiology, 119992, Moscow, Russia

**Keywords:** Disseminated tuberculosis, Multisystem involvement, Immunocompetent host, Genitourinary tuberculosis, Multimodal imaging, Histopathological confirmation, Multidisciplinary management, Extrapulmonary TB, Peritoneal tuberculosis, Adnexal mass mimic, FDG-PET/CT, Spondylodiscitis, Histopathology, Biopsy-guided diagnosis, SDG 3-good health and well-being, SDG 4-quality education, SDG 9-industry, Innovation and infrastructure, SDG 17-partnerships for the goals

## Abstract

**Background:**

Disseminated tuberculosis (dTB) can occur in immunocompetent adults, frequently mimicking metastatic malignancy, thereby delaying the diagnosis.

**Case Presentation:**

A young woman without known immunosuppression developed multisystem disease involving the peritoneum/ovaries, hepatobiliary structures, lymph nodes, adrenals, and thoracolumbar spine. CT/MRI and PET/CT suggested widespread neoplastic disease. Because FDG avidity is nonspecific, we prioritized histologic confirmation. Surgical exploration and targeted biopsies showed necrotizing granulomatous inflammation compatible with tuberculosis; microbiologic testing supported the diagnosis. The patient commenced directly observed first-line therapy (isoniazid, rifampin, pyrazinamide, ethambutol) as the intensive phase, followed by an isoniazid-rifampin continuation phase. Under treatment, symptoms improved, and interval imaging showed regression of inflammatory lesions.

**Conclusion:**

In cancer-like, multisystem presentations, even in apparently immunocompetent hosts, tissue diagnosis is decisive, and imaging should primarily guide sampling. Early recognition and standardized therapy can prevent irreversible morbidity.

## 1 INTRODUCTION

Tuberculosis (TB) remains a leading infectious cause of morbidity and mortality, with an estimated 10.6 million new cases and 1.6 million deaths in 2022 [1]. While pulmonary TB predominates, extrapulmonary tuberculosis (EPTB) accounts for a substantial minority and is frequently under-recognized; contemporary series place EPTB at ~20–30% of cases. Disseminated TB (dTB), hematogenous or lymphatic spread to two or more noncontiguous organs, remains uncommon, particularly among immunocompetent adults (<~2% of all TB cases) [2-4].

dTB is classically associated with HIV infection, malignancy, or immunosuppression [5]. In immunocompetent individuals, however, it presents infrequently with nonspecific systemic symptoms (fever, weight loss, fatigue, night sweats) and multiorgan involvement that often mimics metastatic malignancy or systemic inflammatory disease, contributing to diagnostic delay [6, 7].

Imaging is central to the initial evaluation. Chest radiographs may show miliary nodules, and CT/MRI often demonstrate necrotic lymphadenopathy, splenic/hepatic microabscesses, and multiorgan granulomatous lesions [6]. Because these patterns overlap with metastatic cancer, lymphoma, and other disseminated infections, FDG-PET/CT is best used to map disease activity rather than to discriminate etiologies; imaging should guide biopsy selection, whereas histopathology and microbiology remain decisive [8-10].

We herein report a diagnostically challenging case of dTB in an immunocompetent woman whose multiorgan imaging simulated metastatic malignancy, underscoring the value of integrating clinical context, targeted imaging, and timely tissue confirmation to achieve precise diagnosis and appropriate therapy [11, 12].

## 2 CASE PRESENTATION

### 2.1 Initial Presentation and Emergency Department Evaluation

A 51-year-old woman presented with abrupt, severe lumbar pain described as burning and radiating to both lower limbs. The episode began during a stand-to-sit transition and escalated within hours, producing functional limitation despite oral analgesics. She was afebrile and hemodynamically stable. Examination showed marked tenderness over the lower thoracic/upper lumbar spine with restricted motion; sensation was preserved, and strength was near normal except for mild L1 weakness. Deep tendon reflexes were symmetric; Bragard’s sign was positive on the right. BMI was 35.1 kg/m^2^ (class I obesity). History included antiphospholipid syndrome (APS) treated with therapeutic enoxaparin.

### 2.2 Relevant Medical History Prior to Admission

Five months earlier, the woman sustained a T12 compression fracture after a mechanical fall; persistent pain led to percutaneous vertebroplasty two months later without immediate complications. One month before presentation, urogenital tuberculosis was diagnosed at a tertiary center, and directly observed HRZE (local program “DOTBAL”) was initiated (intensive phase) [13]. Her history also included recurrent venous thromboembolism, bilateral DVTs (3-4 years earlier), and a PE (2 years earlier), the basis for long-term anticoagulation. Prior procedures included abdominoplasty with liposuction and breast implants (>10 years before). One year earlier, she received two units of packed red blood cells for a gynecologic hemorrhage (Table **1**).

### 2.3 Initial Imaging and Diagnostic Workup

**Fig. (1):**
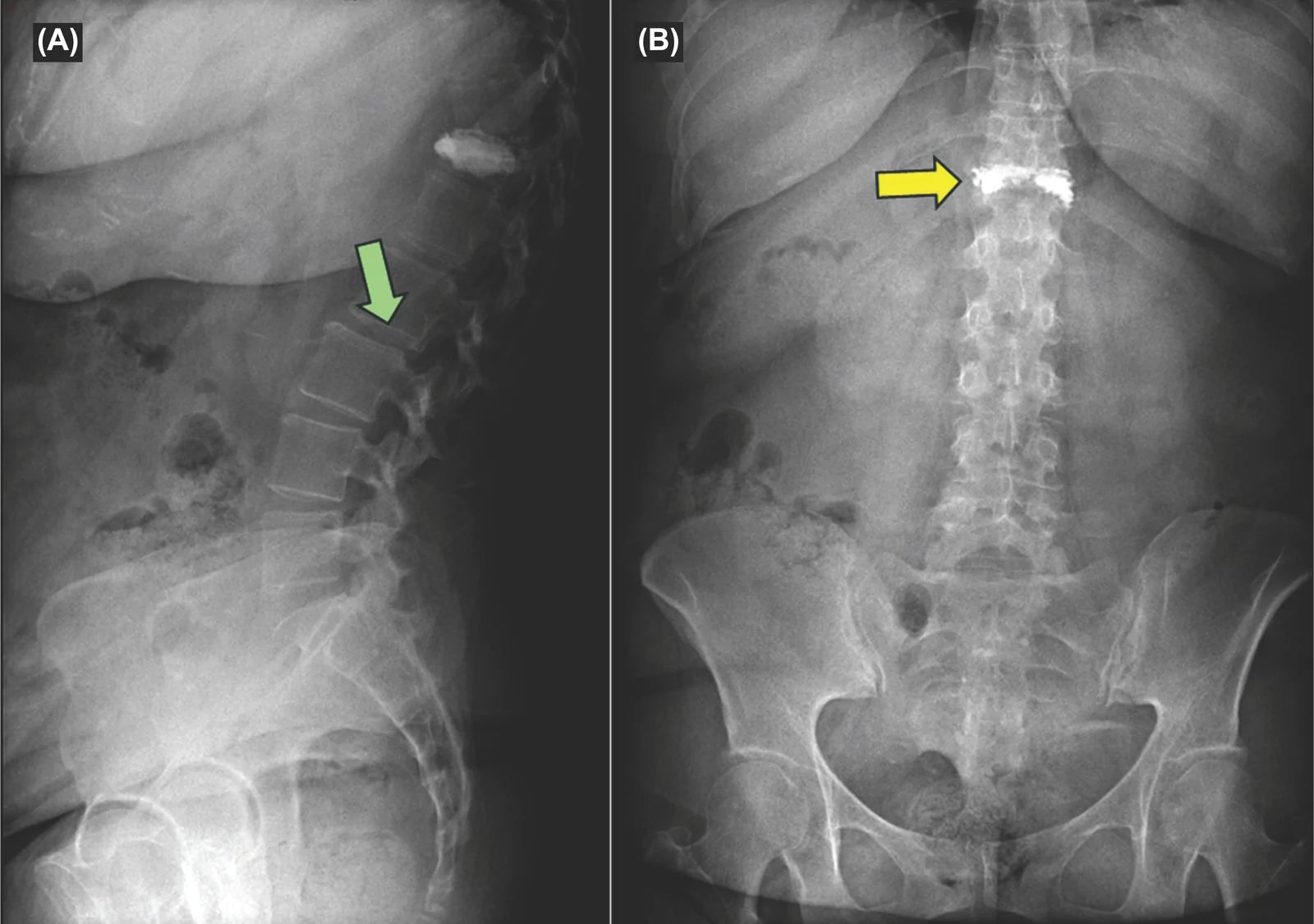
Lumbar spine radiographs [AP (**A**), lateral (**B**)]. Reduced T12 vertebral height with hyperdense vertebroplasty cement (yellow arrow) extending toward the left T12 neural foramen; mild subchondral sclerosis and early anterior osteophytes. L2 on L3 posterior displacement <25% (Meyerding grade I, green arrow). Increased thoracic kyphosis. Facet joints and posterior elements are preserved. **Abbreviation:** AP, anteroposterior.

**Fig. (2):**
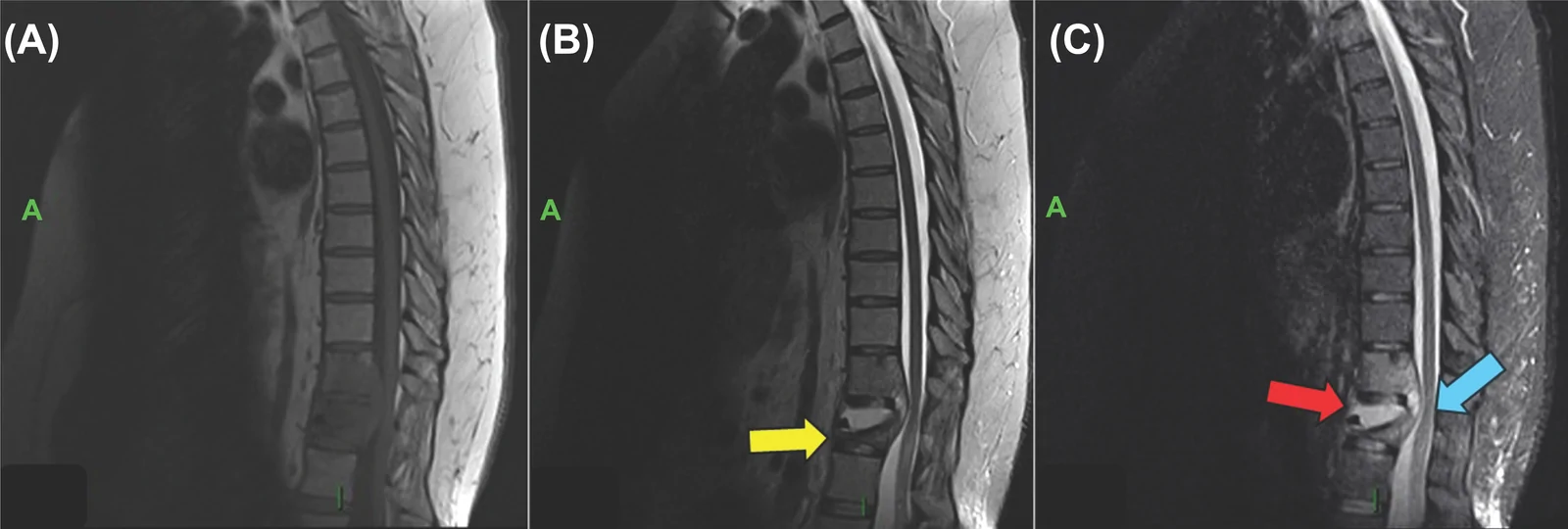
Thoracolumbar MRI demonstrating sagittal, T1-weighted (**A**), T2-weighted (**B**), STIR (**C**). Marked T12 height loss with anterior wedging (yellow arrow) and cement from prior vertebroplasty (red arrow). Epidural phlegmon/abscess produces ≈60% canal narrowing with features of compressive myelopathy (blue arrow). **Abbreviations:** MRI, magnetic resonance imaging; STIR, short-tau inversion recovery.

**Fig. (3):**
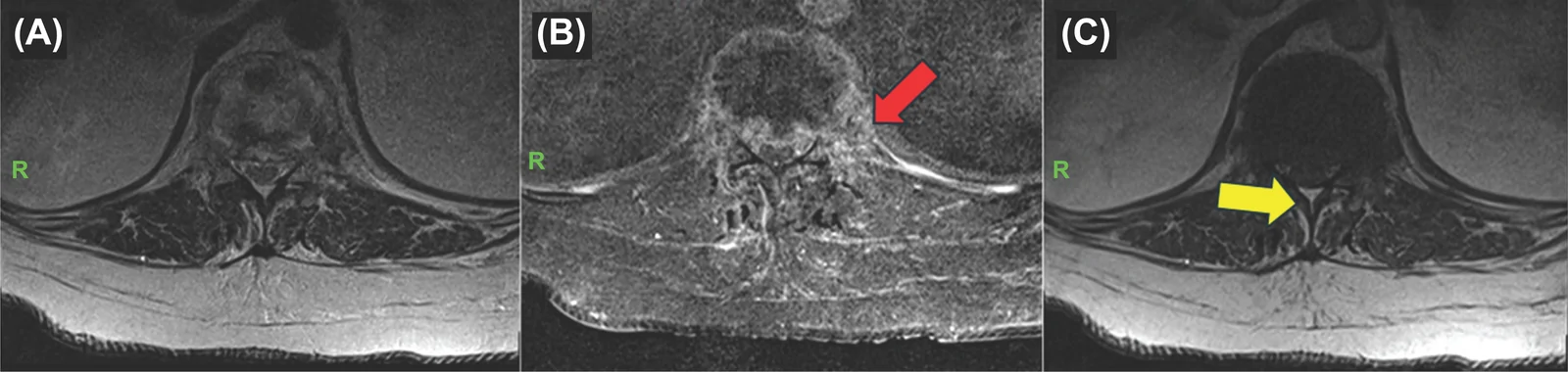
Thoracolumbar MRI at T12, axial T1-weighted (**A**), post-gadolinium T1-weighted (**B**), T2-weighted (**C**). Hypointense marrow signal in T12 (**A**, **C**) with intense post-contrast enhancement (**B**). Subligamentous collection (red arrow) contributes to ≈60% spinal stenosis and compressive myelopathy (yellow arrow). **Abbreviation:** MRI, magnetic resonance imaging.

Thoracolumbar radiographs at admission (Fig. **1**) showed hyperdense vertebroplasty cement within T12 with partial extension into the left T12 neural foramen, accentuating local kyphosis; no acute fracture was seen. On hospital day 1, contrast-enhanced MRI (Figs. **2**, **3**) demonstrated tuberculous spondylodiscitis at T11–T12: disc and adjacent vertebral involvement with an epidural soft-tissue component bulging the posterior longitudinal ligament and causing approximately 60% canal narrowing. The woman was admitted for multimodal analgesia and coordinated infectious-disease and neurosurgical care.

### 2.4 Hospital Week 1: Acute Urological Complications

**Fig. (4):**
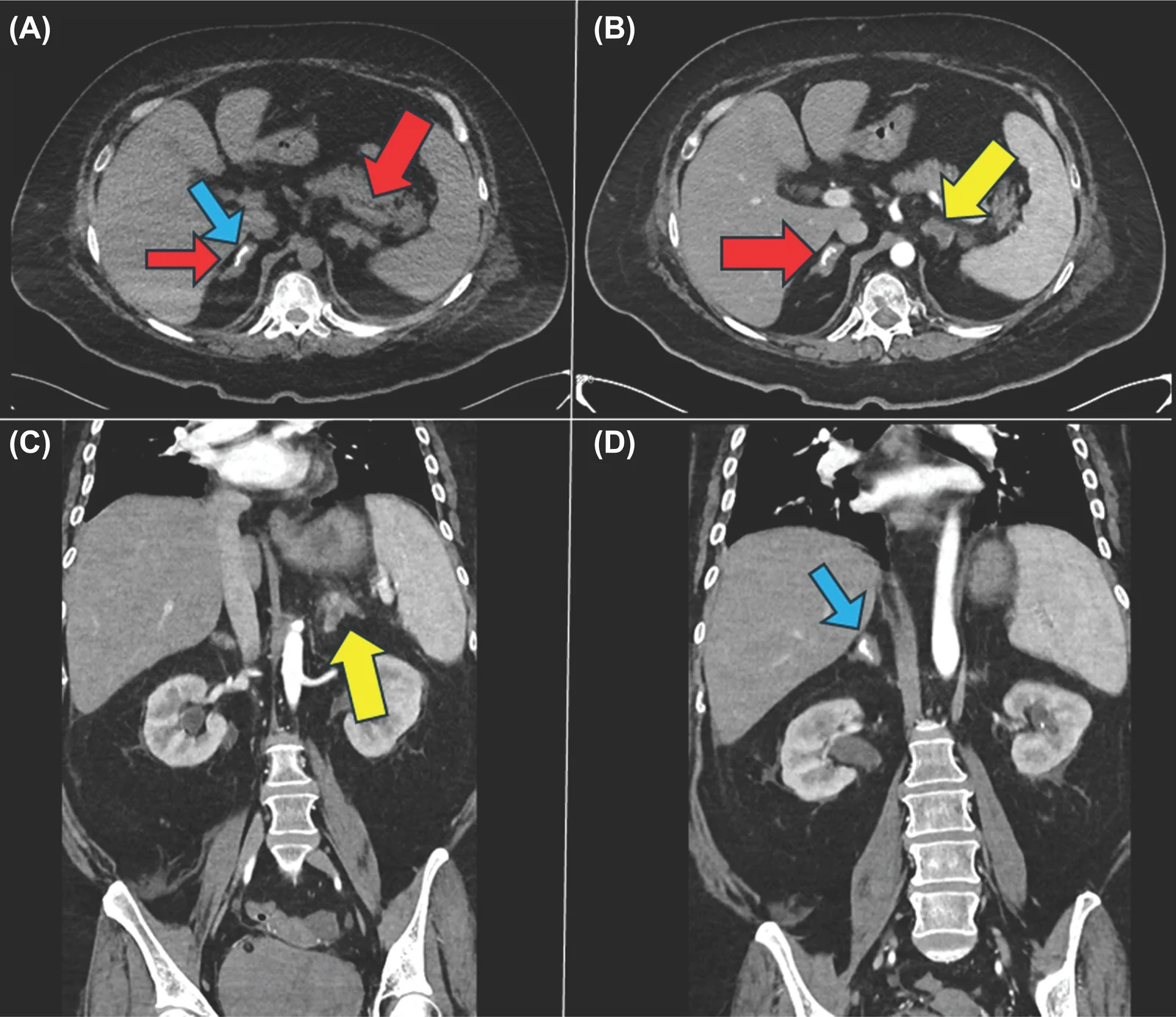
Abdominal CT showing non-contrast axial (**A**), contrast-enhanced axial (**B**), and coronal reconstructions (**C**, **D**). Bilateral adrenal hypertrophy (red arrows) with right-sided calcifications (blue arrow) and areas of reduced contrast enhancement/hypoperfusion (yellow arrows). **Abbreviation:** CT, computed tomography.

**Fig. (5):**
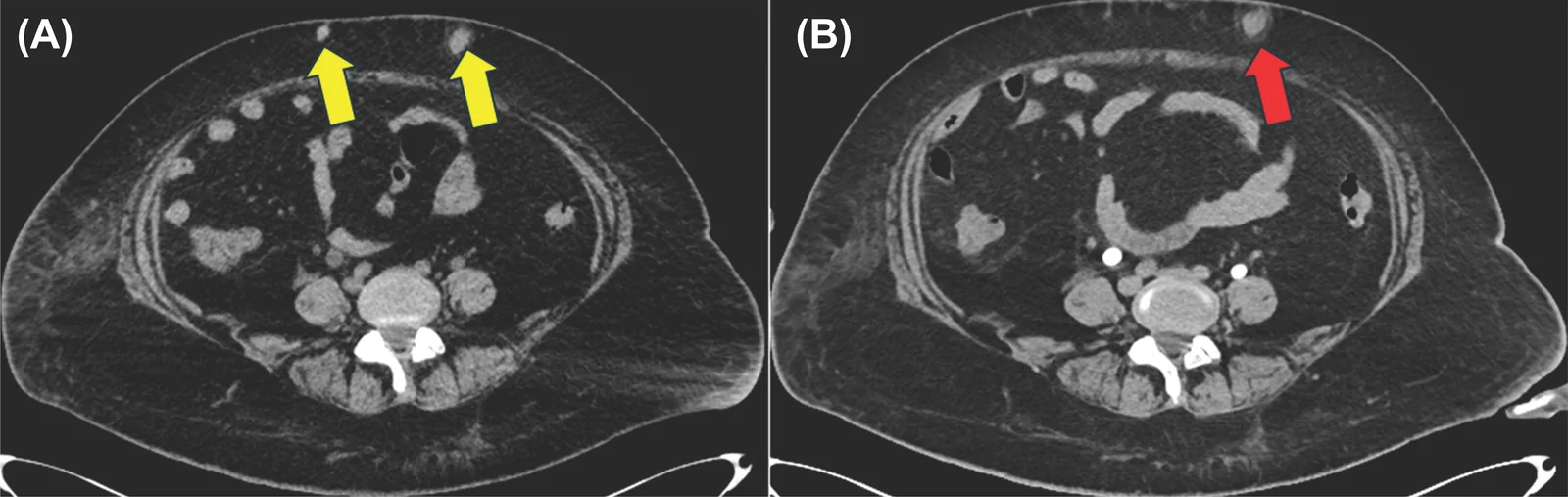
Axial abdominal CT non-contrast (**A**) and contrast-enhanced images (**B**). Hyperdense anterior abdominal-wall lesions with hypodense centers (yellow arrows), with some containing air pockets; the largest lesion (17 mm) lacks post-contrast enhancement (red arrow), consistent with granulomatous nodules. **Abbreviation:** CT, computed tomography.

**Fig. (6):**
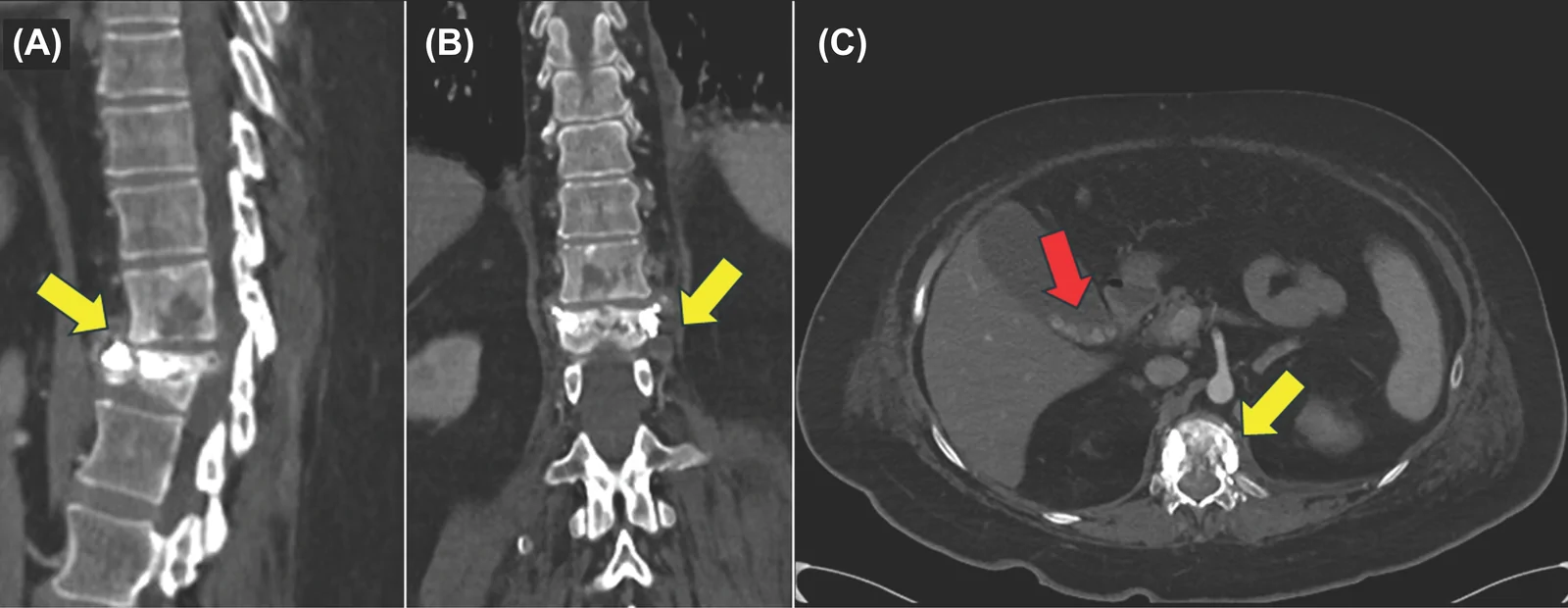
Abdominopelvic CT sagittal (**A**), coronal (**B**), and axial images (**C**). T12 insufficiency fracture with cement *in situ* (yellow arrow). Multiple hyperdense gallstones compatible with cholelithiasis (red arrow). **Abbreviation:** CT, computed tomography.

**Fig. (7):**
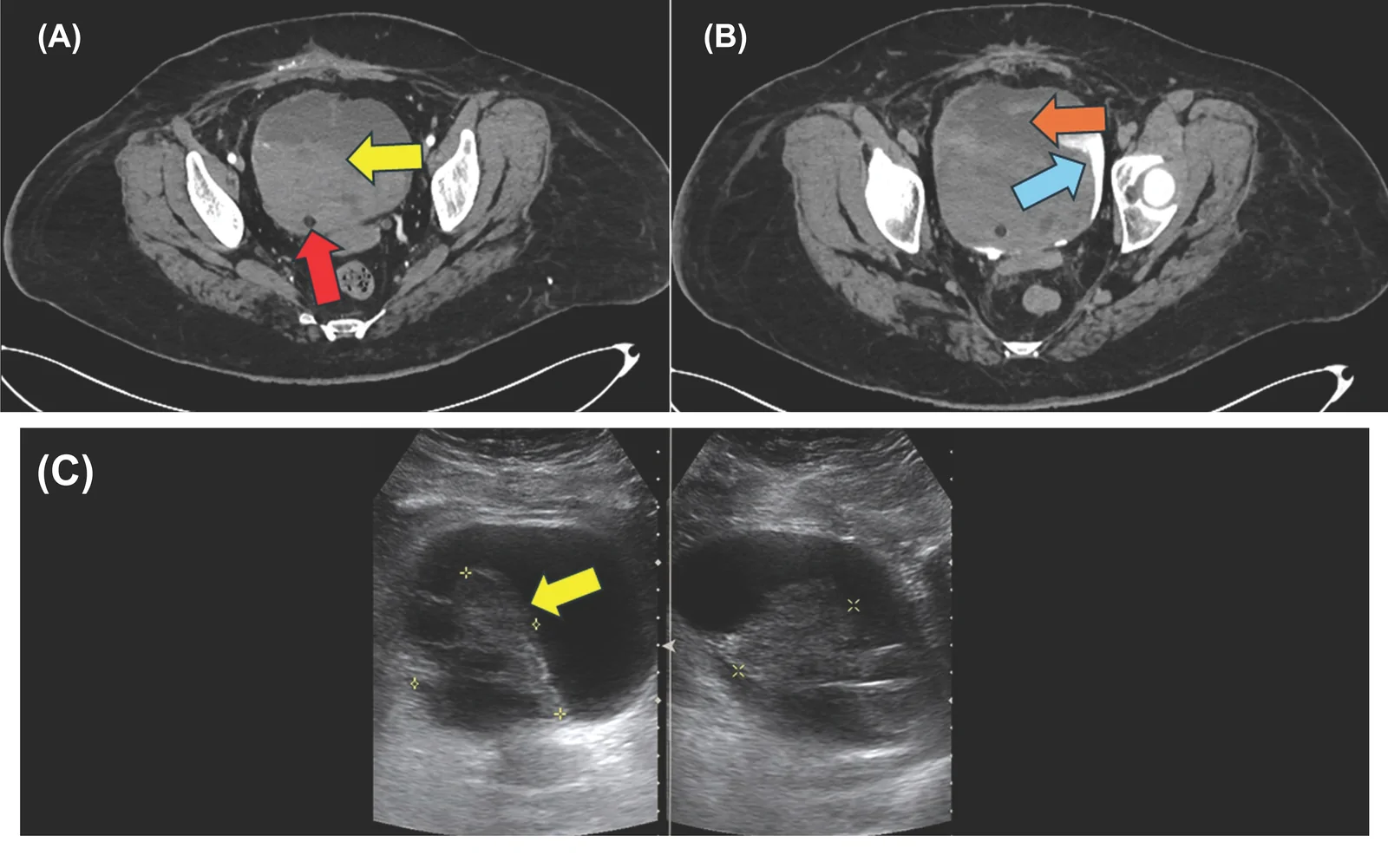
Bladder imaging CT and ultrasound (US). Non-contrast CT (**A**) and excretory-phase CT (**B**) show a distended bladder with a Foley catheter (red arrow), hyperdense clots ~48 HU (yellow arrow), and filling defects ~18 HU (orange arrow) compatible with hematic/granulomatous material. US (**C**) demonstrates an echogenic mobile intravesical mass causing incomplete emptying. **Abbreviations:** CT, computed tomography; US, ultrasound; HU, Hounsfield units.

By day 3, attention shifted to gross hematuria with clot retention and painful bladder distension; anticoagulation was temporarily held. On day 4, contrast-enhanced CT urography (Figs. **4**, **5**) revealed bilateral hypodense renal parenchymal lesions compatible with tuberculous involvement, perinephric fat stranding, left proximal ureteral calculus (~5 mm), and bilateral adrenal enlargement with right-sided calcifications and areas of hypoperfusion. Bone windows confirmed prior T12 insufficiency fracture and cement *in situ*; cholelithiasis was incidentally noted (Fig. **6**). A large intravesical clot occupied the bladder lumen (Fig. **7**).

Continuous three-way irrigation was initiated. Ongoing obstruction prompted operative cystoscopy with bipolar transurethral evacuation of a large, organized clot. Hemoglobin fell to 6.9 g/dL intraoperatively; one PRBC unit was transfused with a good response. Bladder-clot and urine specimens were sent for mycobacterial/bacterial cultures and PCR. Empiric meropenem plus linezolid was started for possible superinfection while antituberculous therapy continued. A pain-medicine consult optimized multimodal analgesia. By the end of the week, hematuria had resolved, and anticoagulation was cautiously resumed.

### 2.5 Hospital Week 2: Multidisciplinary Care and Hematologic Management

Urine GeneXpert PCR (≈day 6) detected *M. tuberculosis* DNA, corroborating genitourinary TB. A milder hematuria recurrence required a second cystoscopic evacuation; follow-up bladder ultrasound documented ~75 mL clot clearance (Fig. **7**). Small pleural effusions responded to intravenous diuretics and pulmonary-rehabilitation exercises.

For analgesia, an erector spinae plane (ESP) catheter at T11 provided continuous regional blockade with intermittent ropivacaine boluses, supplemented by intravenous buprenorphine, oral pregabalin, and acetaminophen, improving the mobility. Hematologic monitoring showed normocytic anemia (nadir 6.4 g/dL) and leukopenia; linezolid was discontinued for suspected marrow suppression. Management included intravenous ferric carboxymaltose, high-dose erythropoietin, folate, and transfusional support, with gradual recovery. The patient remained afebrile and clinically stable.

### 2.6 Hospital Week 3: Spinal Surgery and Postoperative Course

Persistent T11-T12 cord compression, mechanical instability, and refractory pain prompted surgery: T11-T12 laminectomy with canal decompression, debridement of infected/necrotic tissue, partial facetectomies/flavectomies, and posterior instrumentation from T10-L2. The procedure was uneventful.

**Fig. (8):**
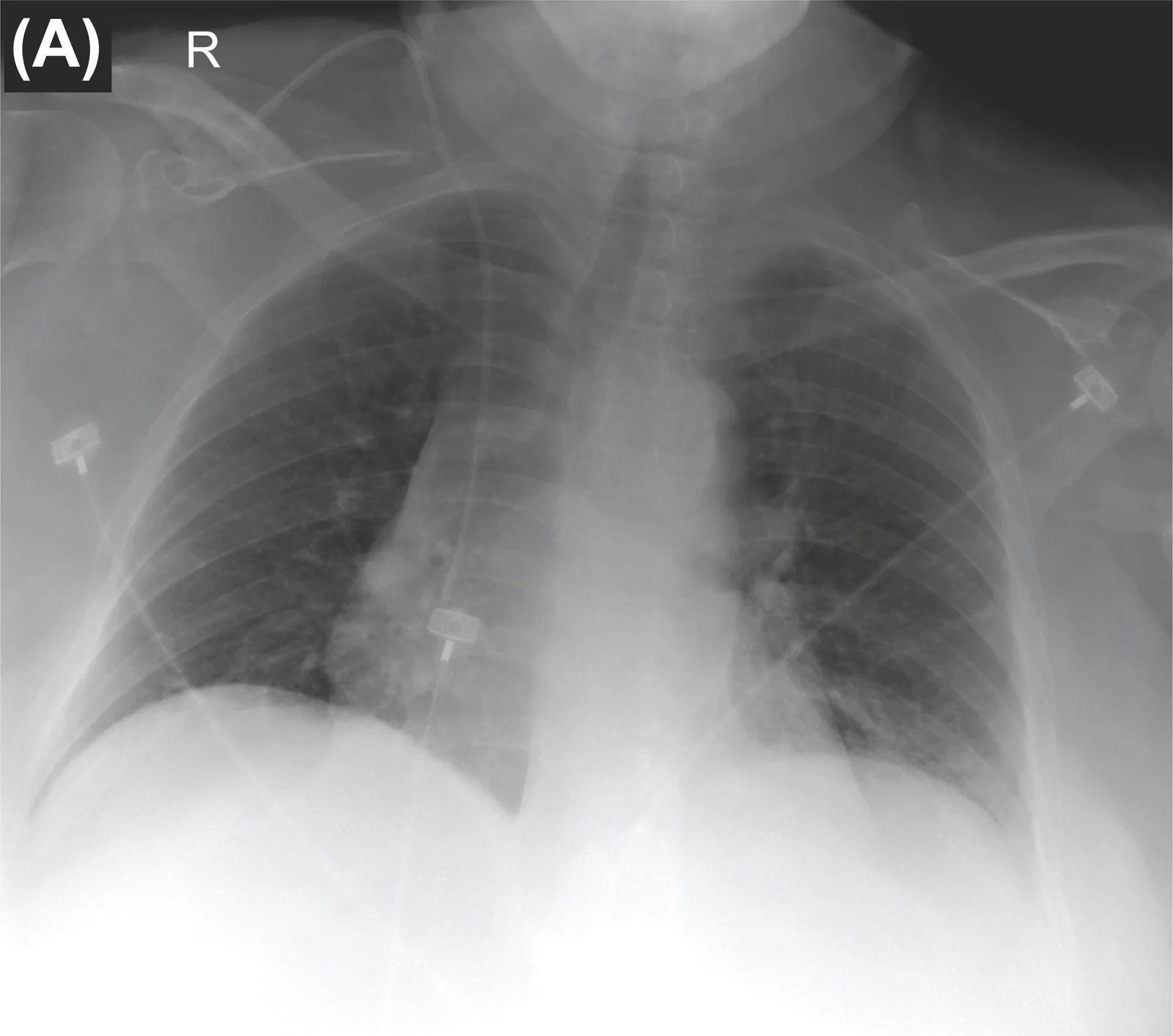
Chest radiograph. Bilateral hypoventilation with left basal reticular opacities and a small left pleural effusion; an indwelling central venous catheter *in situ*.

**Fig. (9A-C):**
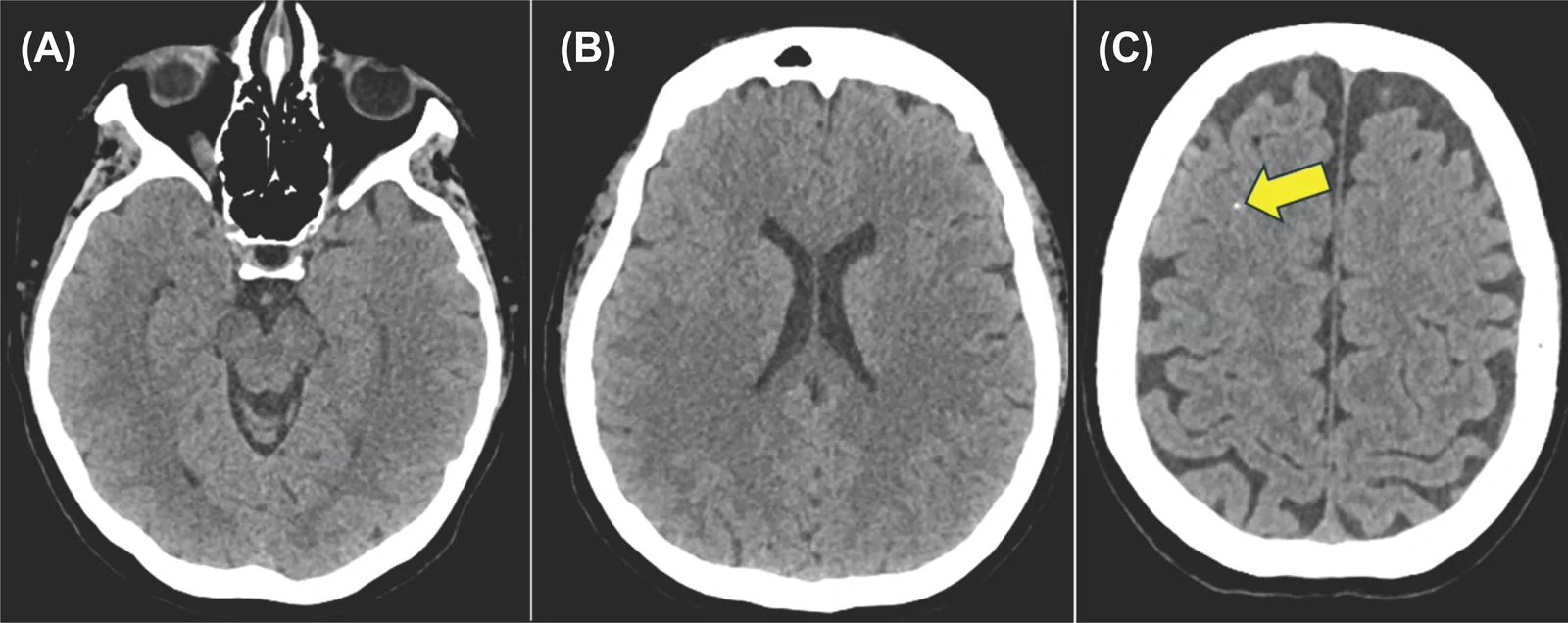
Non-contrast head CT. Multiple small cortico-subcortical calcifications in both hemispheres (yellow arrow: right hemisphere). No acute hemorrhage or mass effect. **Abbreviation:** CT, computed tomography.

In the immediate post-operative period, mild wheeze and exertional dyspnea, attributed to basal atelectasis/airway irritation, resolved with inhaled bronchodilators and chest physiotherapy; chest radiography showed bilateral hypoventilation, a left basal reticular pattern, and a small effusion (Fig. **8**). Around the same time, transient vertigo occurred; non-contrast head CT demonstrated bilateral cortico-subcortical calcifications without hemorrhage or mass effect (Fig. **9**).

### 2.7 Hospital Weeks 4-5: Wound Infection and Ongoing Management

**Fig. (10):**
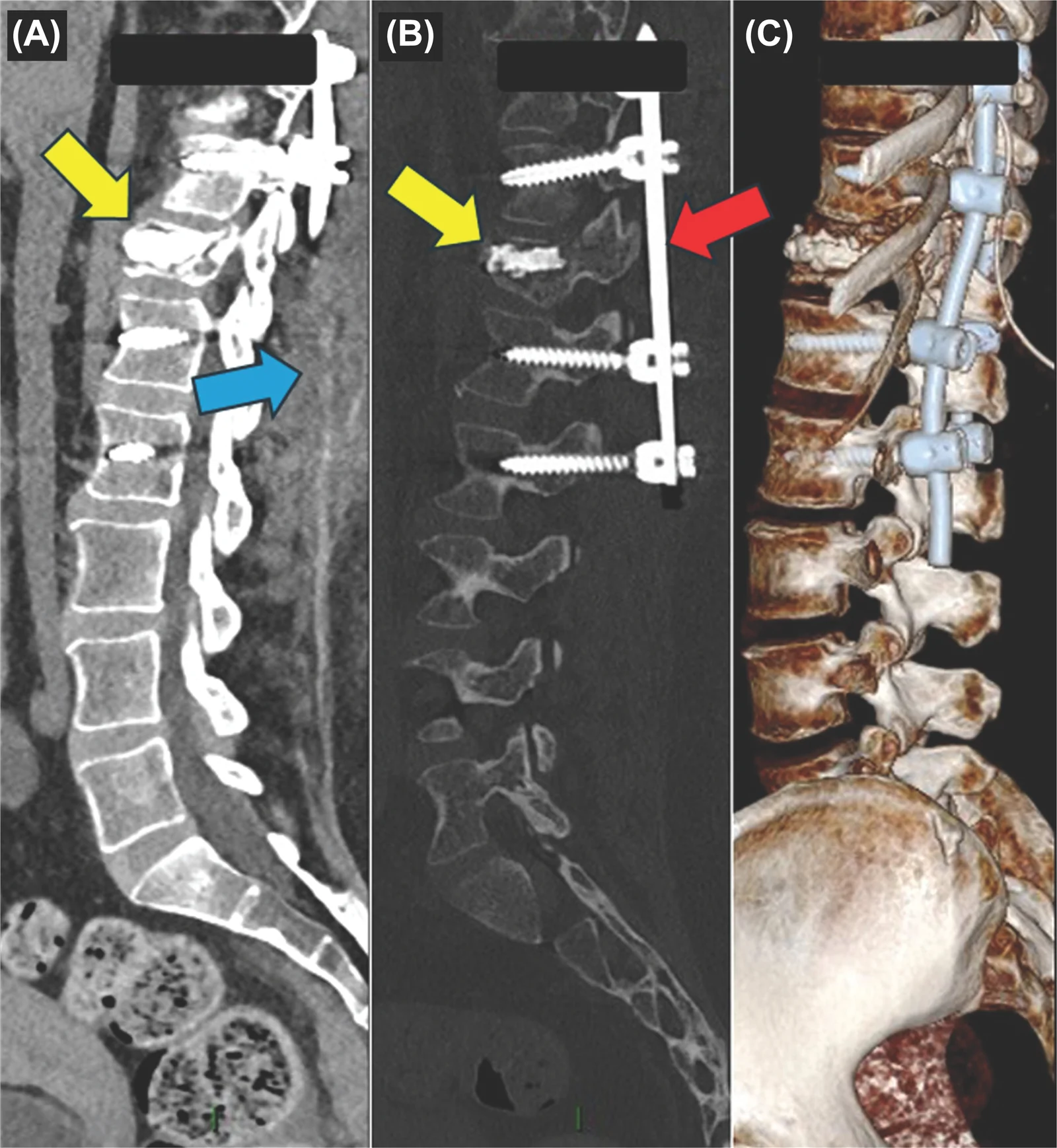
Lumbar spine CT showing sagittal soft-tissue (**A**), sagittal bone (**B**), and 3D rendering (**C**). Post-operative transpedicular screws at T10, T11, L1, and L2 (red arrows). Posterior decompression changes at T11–T12, including laminectomy, hemifacetectomy, drain in place (blue arrows). Cementoplasty at T12 (yellow arrow). Relative loss of lumbar lordosis. **Abbreviations:** CT, computed tomography; 3D, three-dimensional.

During week 4, CT thoracolumbar spine identified hardware complications: T12 pedicle fracture, displacement of T11/T12 screws, and partial migration of one screw into the canal, with bilateral pleural effusions and left lower-lobe atelectasis (Fig. **10**). Given anemia and overall fragility, the team deferred immediate revision and pursued medical optimization.

**Fig. (11):**
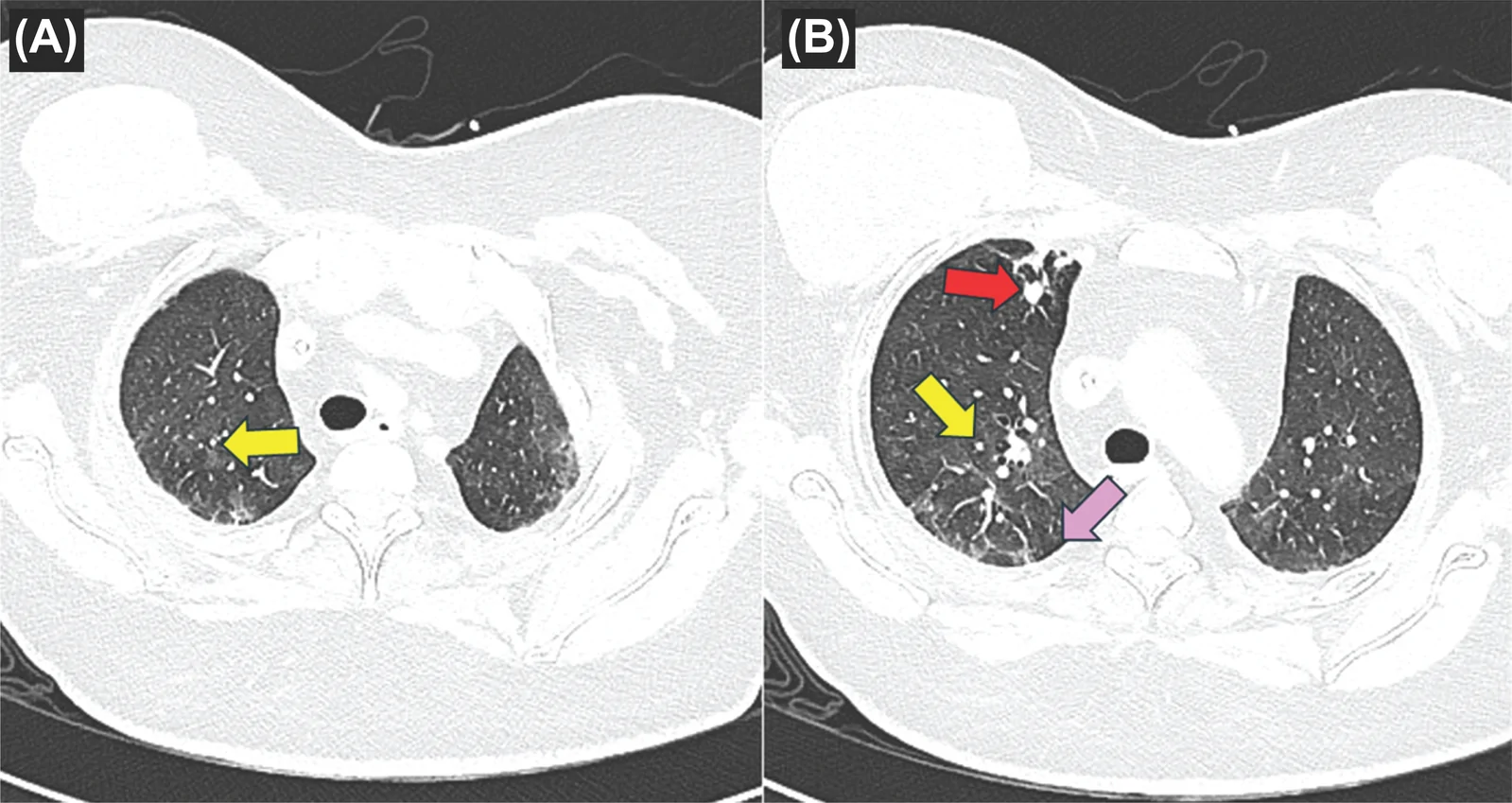
Chest CT, lung window (**A**, **B**). Ground-glass opacities in upper lobes and upper segments of lower lobes (yellow arrows); 1-cm cavitary nodule in the right upper-lobe apical segment (red arrow); subsegmental atelectasis (purple arrow). **Abbreviation:** CT, computed tomography.

**Fig. (12):**
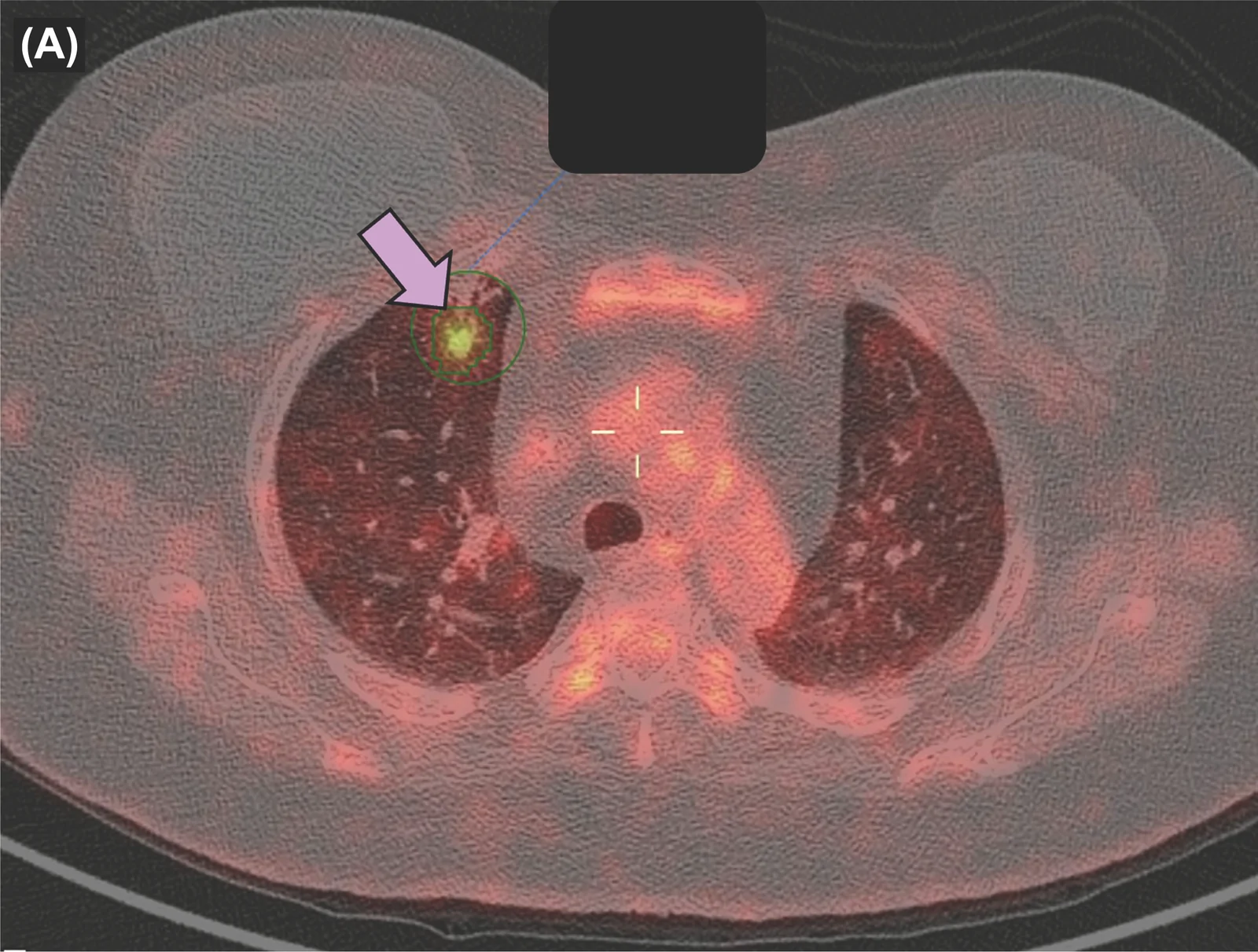
18F-FDG PET/CT. Hypermetabolic spiculated pulmonary nodule in the right upper lobe with SUVmax 4.0 (purple arrow) and local architectural distortion. **Abbreviations:** FDG, fluorodeoxyglucose; PET/CT, positron emission tomography/computed tomography; SUVmax, maximum standardized uptake value.

**Fig. (13):**
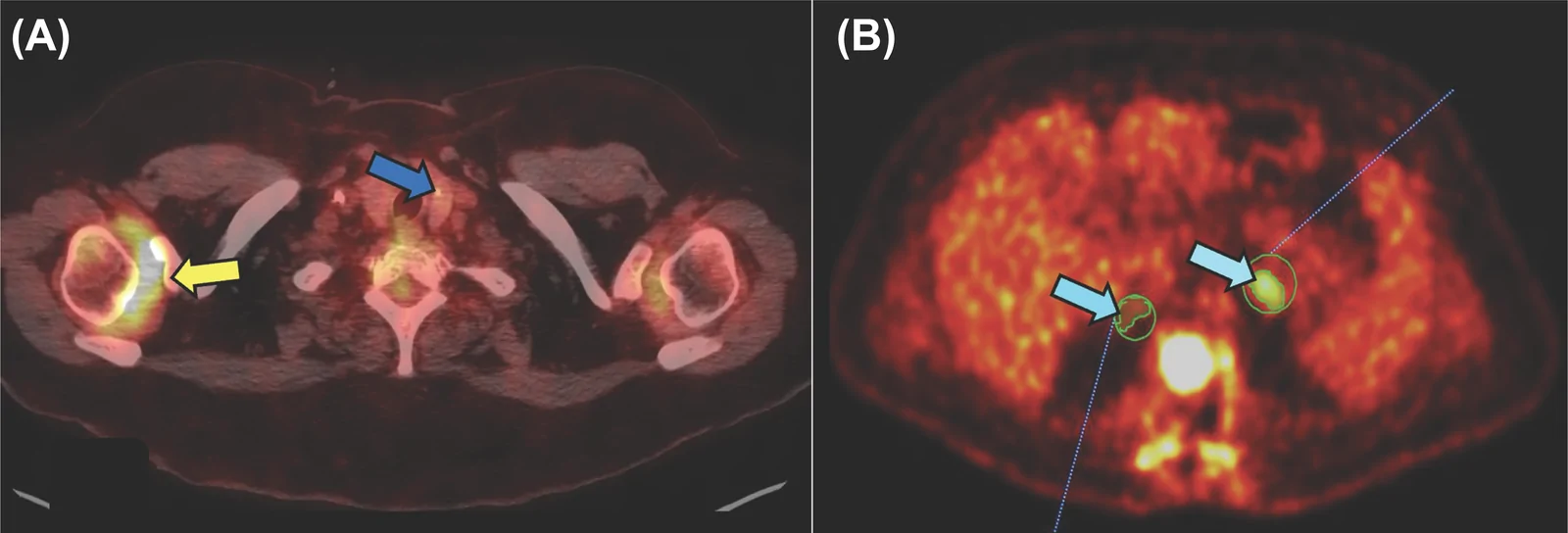
18F-FDG PET/CT. (**A**) Right shoulder with synovial thickening/effusion and diffuse uptake (SUVmax 7.6), suggestive of synovitis (yellow arrow). Left thyroid nodule with increased uptake (dark-blue arrow). (**B**) Bilateral adrenal thickening with nodular morphology and right-sided calcification; intense uptake (SUVmax 5.9) (light-blue arrows). **Abbreviations:** FDG, fluorodeoxyglucose; PET/CT, positron emission tomography/computed tomography; SUVmax, maximum standardized uptake value.

^18^F-FDG PET/CT showed a hypermetabolic spiculated right-upper-lobe pulmonary nodule (Figs. **11**, **12**), bilateral adrenal uptake with nodular thickening and calcifications (right-predominant), a hypermetabolic left thyroid nodule, low-grade uptake in subcutaneous abdominal-wall nodules, and right-shoulder uptake suggesting synovitis (Fig. **13**).

Two weeks after instrumentation, increasing serosanguineous drainage with surrounding erythema/induration suggested an early deep surgical-site infection. The patient underwent surgical debridement with copious irrigation and VAC placement. Cultures demonstrated the growth of *Staphylococcus epidermidis* (wound) and ESBL *Escherichia coli* (urine). Antibiotic therapy was limited to intravenous vancomycin and meropenem, and the urinary catheter was removed.

By the end of week 5, the patient was alert, fully oriented, and without focal deficits or recurrent vertigo. Lower-extremity strength and sensation were preserved; assisted ambulation progressed as pain and segmental stability improved. The wound under VAC therapy showed healthy granulation without purulence; delayed closure was planned once the bed matured. Antituberculous therapy transitioned to the continuation phase under infectious-disease supervision (Tables **1**–**3**).

### 2.8 Discharge Status and Follow-up

**Fig. (14):**
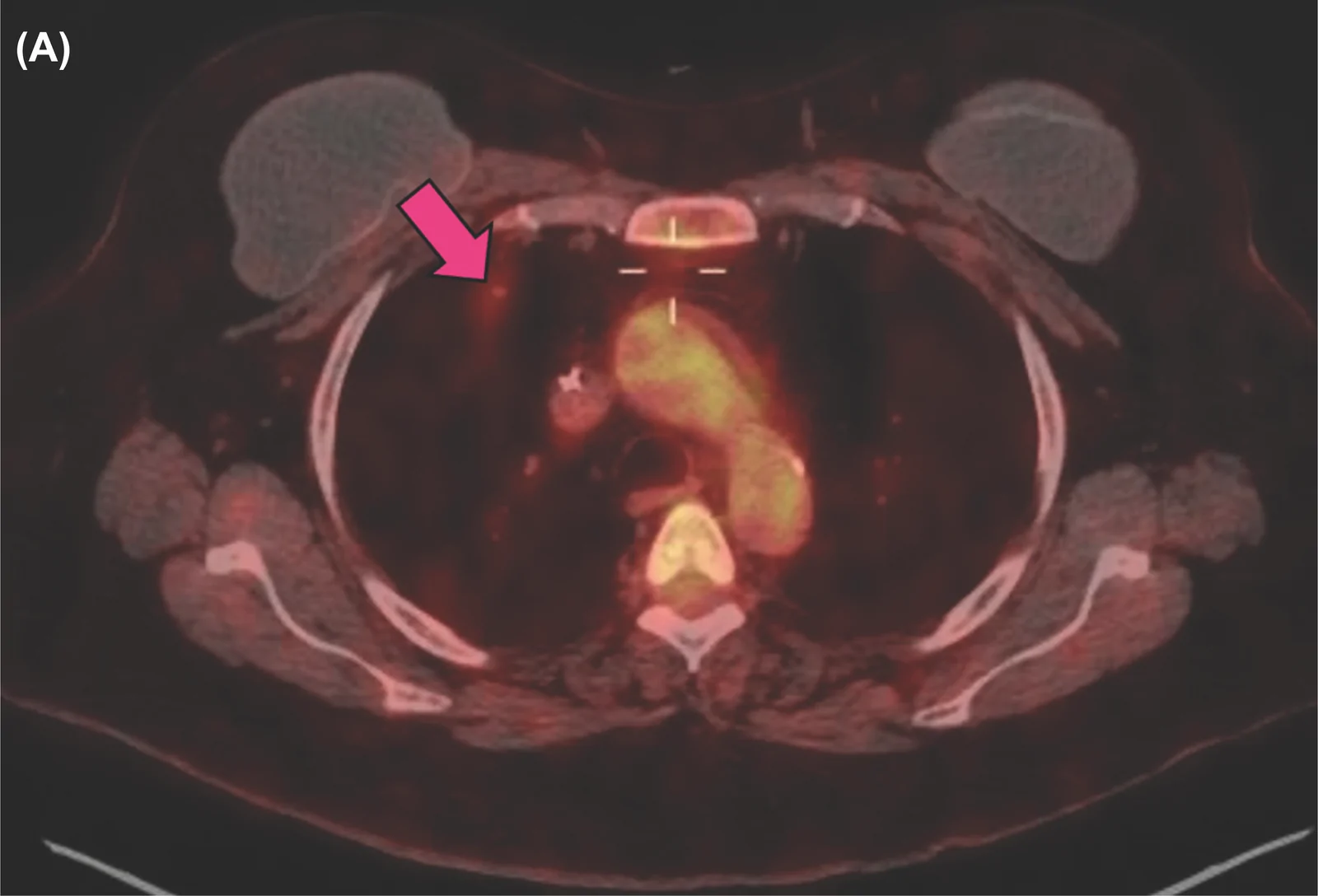
18F-FDG PET/CT (~3-month follow-up). Decreased size and metabolic activity of the previously noted right upper-lobe pulmonary nodule (pink arrow), consistent with interval treatment response. **Abbreviations:** FDG, fluorodeoxyglucose; PET/CT, positron emission tomography/computed tomography; SUVmax, maximum standardized uptake value.

After >5 weeks, the patient was discharged clinically stable and neurologically intact, with mild incision pain controlled by oral analgesics. Early post-discharge PET/CT documented decreased size and metabolic activity of the right-upper-lobe nodule (Fig. **14**), indicating interval response. She continued the continuation-phase antituberculous regimen with scheduled laboratory monitoring and interval imaging to confirm disease resolution and assess instrumentation integrity prior to any planned hardware revision.

## 3 DISCUSSION

### 3.1 Atypical Disseminated Tuberculosis in an Immunocompetent Host

Although tuberculosis is classically linked to immunocompromised states, disseminated TB (dTB) also occurs in immunocompetent adults, reflecting nuanced host-pathogen interactions [5, 14, 15]. Our patient, without HIV or recognized immunosuppression, illustrates this scenario. Host factors (*e.g*., toll-like receptor/cytokine polymorphisms) may blunt early containment of *Mycobacterium tuberculosis* and permit hematogenous spread [6, 10]; hypervirulent lineages, such as Beijing, have likewise been associated with broader dissemination even in the absence of traditional risks [16].

Radiologically, dTB frequently mimics metastatic cancer, contributing to delay. In this case, multiorgan involvement (peritoneum/ovaries, hepatobiliary system, lymph nodes, adrenals, thoracolumbar spine) created a malignant-appearing pattern on cross-sectional imaging. PET/CT was useful for mapping burden and selecting biopsy targets, but insufficiently specific to exclude malignancy [9, 17-19]. Gynecologic-peritoneal TB can be indistinguishable from ovarian carcinoma on imaging and tumor markers, often necessitating diagnostic laparoscopy/laparotomy with histology [20-22]. Accordingly, tissue diagnosis remained decisive and justified surgical exploration/biopsy in our patient [10].

### 3.2 Implication for Practice

In endemic settings, and in cancer-like multisystem presentations, even among apparently immunocompetent hosts, a high index of suspicion for disseminated tuberculosis should be maintained. A pragmatic sequence should include the following: **(1)** CT/MRI to define anatomic extent; **(2)** PET/CT to select metabolically active targets for sampling [9, 17, 18]; and **(3)** histopathologic confirmation with mycobacterial testing [10]. Early recognition and prompt therapy can help prevent irreversible morbidity.

### 3.3 Multisystem Involvement and Diagnostic Complexity

This patient’s course involved neurological, gastrointestinal, genitourinary, and musculoskeletal systems, underscoring TB’s capacity for multisystem dissemination. Severe headaches prompted early neuroimaging, consistent with clinical features associated with increased morbidity in TB meningitis [14, 15]. Peritoneal involvement with lymphadenopathy and tuberculomas on CT/MRI in an immunocompetent host is uncommon, contributing to delay [23, 24].

A recent series of immunocompetent individuals with multiorgan TB reported similar challenges: systemic disease was confirmed only after advanced imaging identified occult foci [25, 26]. Whole-body approaches (*e.g*., PET/CT and comprehensive MRI) can detect subclinical organ involvement, such as renal or peritoneal TB, which conventional imaging may miss [4].

These observations suggest that dTB in immunocompetent hosts may follow non-classical patterns, shaped by pathogen virulence and host immune variability. In our case, the disease trajectory, initially musculoskeletal, then involving the genitourinary and intra-abdominal systems, mirrored prior reports in which the full extent of disease was revealed only through multi-modality imaging [15, 27]. Recognizing such atypical distributions is crucial for timely therapy, even when overt immunosuppression is absent.

### 3.4 Surgical Findings and Histopathology in Disseminated TB

Surgery in this case yielded both therapy and diagnosis. Macroscopic features, including peritoneal thickening, caseating nodules, and mass-like lesions, are well known to mimic malignancy, explaining the need for operative sampling when imaging is equivocal [10, 23]. Post-spinal-surgery care with a vacuum-assisted closure (VAC) system aided drainage and granulation in a high-burden inflammatory setting [28, 29].

Despite rapid molecular assays, histopathology remains a cornerstone of TB diagnosis, particularly with low bacillary load or pending/negative cultures [6, 14]. Even when molecular tests confirm TB, contemporary data emphasize that tissue examination provides indispensable clarity for extrapulmonary and disseminated disease, especially in atypical presentations [3, 30]. In our patient, surgical exploration and tissue analysis confirmed the diagnosis and directed targeted antimicrobial and surgical planning, underscoring the value of coordinated multidisciplinary care in complex dTB.

### 3.5 Radiologic Clues and Imaging-driven Diagnosis

Imaging was central to mapping disease and guiding decisions. Contrast-enhanced CT and MRI showed peritoneal thickening, necrotic lymphadenopathy, and intracranial calcifications compatible with tuberculomas, findings consistent with reports highlighting advanced imaging for subtle or atypical TB [15, 27, 31]. MRI defined the extent of tuberculous spondylodiscitis, while PET/CT captured whole-body activity and later documented interval response (Table **3**; Fig. **14**). Because dTB can closely mimic malignancy or immune-mediated disease, including mass-like enhancement, central necrosis, confluent nodes, and peritoneal caking, imaging should guide biopsy selection, whereas histopathology/microbiology may provide the definitive diagnosis. Emerging tools, including radiomics and AI classifiers, may facilitate TB-neoplasia differentiation in complex presentations [17, 32, 33]. At present, they are adjuncts, not replacements, for tissue confirmation.

### 3.6 Clinical Evolution and Treatment Response

Despite early deterioration and multisystem involvement, the patient improved after initiation of first-line antituberculous therapy together with targeted surgical and supportive care. During admission, vital signs stabilized, gross hematuria resolved, neurologic function was preserved, and organ-specific abnormalities regressed with treatment (Tables **1**, **2**). An early post-discharge PET/CT demonstrated decreased size and FDG uptake of the right-upper-lobe nodule, confirming interval response (Fig. **14**).

Adjunctive corticosteroids or other immunomodulators, often considered for CNS disease or IRIS (immune reconstitution inflammatory syndrome), were not required in this case [10]. Although IRIS has been reported in 2-23% of patients starting anti-TB therapy, mainly among HIV-positive populations, it appears uncommon in immunocompetent hosts [34, 35]; avoidance of this complication likely contributed to the patient’s steady trajectory.

Overall, this experience shows that disseminated TB with mass-like lesions and multiorgan dysfunction can respond well to standard first-line regimens when diagnosis is timely and management is coordinated [4, 36].

### 3.7 Lessons Learned and Public Health Considerations

Disseminated TB presenting with vague, organ-specific symptoms in an immunocompetent adult often leads to diagnostic delays. In this case, the insidious multisystem pattern, encompassing spinal, renal, adrenal, and pulmonary, mimicked malignant and autoimmune phenotypes, reinforcing the need for early biopsy-directed confirmation and coordinated multidisciplinary care (infectious disease, spine surgery, hematology, pulmonology, urology, rehabilitation).

At the population level, pandemic-related disruptions in screening, access, and surveillance have amplified underdiagnosis and treatment delays [24, 37, 38]. Sustained vigilance for atypical or extrapulmonary presentations, streamlined diagnostic pathways, and structured follow-up are essential to reduce preventable morbidity.

### 3.8 Key Clinical Insights

- **Disseminated TB can Occur in Immunocompetent Hosts:** The absence of classical immunosuppression does not exclude dTB, especially with multiorgan involvement [3, 9, 10].
- **Radiology and Surgical Findings may Mimic Malignancy, but Biopsy Remains Decisive:** Spiculated pulmonary nodules, necrotic lymphadenopathy, peritoneal caking, and adrenal hypermetabolism often resemble neoplasia; imaging should guide the biopsy target, while histopathology/microbiology may confirm the diagnosis [13, 14].
- **Spinal TB Threatens Mechanical Stability and Neurologic Function:** Early spine-surgery input is critical when vertebral involvement and canal compromise are present, even after vertebral augmentation, to avoid irreversible deficits (Figs. **2**, **3**, **10**).
- **Multidisciplinary Coordination is Essential:** The outcomes improve when infectious diseases, spine surgery, hematology, urology, pulmonology, rehabilitation, and pain management teams coordinate procedures, antimicrobial therapy, and supportive care [19, 30].
- **Post-pandemic Surveillance Gaps can Delay Diagnosis:** Reduced screening and disrupted care pathways have increased under-detection, thereby leading to later presentation of TB. Thus, heightened vigilance for atypical/EPTB is warranted [16, 31, 32].

### 3.9 Patient Perspective

The patient expressed satisfaction with the multidisciplinary care received and reported improved quality of life during the follow-up.

## CONCLUSION

Disseminated tuberculosis (dTB) remains difficult to recognize in immunocompetent adults because its multisystem, cancer-like phenotype obscures the infectious cause. In this patient, CT/MRI and PET/CT were used to map burden and target biopsy, and histopathology confirmed dTB, enabling timely, tailored management. Early spine surgery for canal compromise, together with first-line antituberculous therapy and coordinated wound, hematologic, and urologic care, preserved neurologic function and drove recovery.

This case reinforces three practice points: dTB should not be excluded without overt immunosuppression; imaging should be treated as a navigator, and not an arbiter, with tissue confirmation remaining decisive; and care should be coordinated through a multidisciplinary pathway from diagnosis to rehabilitation. Given pandemic-related surveillance gaps, clinicians should maintain heightened vigilance for atypical or extrapulmonary presentations. Early recognition, tissue confirmation, and structured follow-up can convert a complex multisystem illness into a favorable outcome.

## Figures and Tables

**Table 1:** Chronological timeline of major clinical events and interventions (from six months before admission through the end of continuation). This timeline highlights the progression of the patient’s condition and the multidisciplinary management of her disseminated tuberculosis involving the spine and genitourinary tract, as well as associated complications.

**Timepoint (relative to admission)**	**Clinical Events and Exam**	**Imaging/Tests**	**Pathology/Microbiology**	**Treatment/Decision**	**Outcome/Notes**
−6 months	Intermittent abdominal/pelvic pain; weight loss; low-grade fevers	Basic labs non-specific	—	Outpatient workup initiated	Cancer *vs*. infection considered
−3 months	Worsening pain; new back pain	CT abdomen/pelvis: adnexal masses; peritoneal thickening; hepatic lesions	—	Multidisciplinary review recommended	Concern for metastatic malignancy
−1 month	Persistent symptoms	PET/CT: FDG-avid peritoneal, nodal, hepatic, adrenal, and vertebral foci	—	Planned tissue confirmation	Imaging to guide biopsy target
Day 0 (admission)	Evaluation for systemic disease	MRI T-spine: spondylodiscitis at T11-T12 with epidural component	—	Surgical consult	Analgesia; stabilization
Days 3–7	Diagnostic procedures	US/CT-guided sampling of the peritoneal/ovarian/nodal target	Histology: necrotizing granulomas	Initiation of airborne precautions; AFB smear/culture/NAAT	Tissue diagnosis prioritized
Days 10-14	Return of micro results	—	NAAT/culture supportive of TB	Initiation of directly observed HRZE (intensive phase)	Symptomatic improvement
Month 1	Follow-up clinic	CT/MRI for interval assessment	—	Transition plan to HR (continuation phase)	Weight gain; pain improved
Month 3	Imaging reassessment	PET/CT/CT showed decreased metabolic activity and lesion size	—	Continuation of therapy, monitoring of lab results	Continued clinical response
Month 6	End of continuation	CT/MRI demonstrated regression; stable spine	—	Completion of therapy per protocol	No relapse signs; rehabilitation as needed

**Abbreviations:** AFB = acid-fast bacilli; HRZE = isoniazid, rifampicin, pyrazinamide, ethambutol; NAAT = nucleic acid amplification test; PET/CT = positron emission tomography/computed tomography.

**Table 2:** Key laboratory and immunological findings during hospitalization. Values are expressed as absolute measurements with units; reference ranges are shown in parentheses. Timepoints are relative to admission. This table summarizes hematologic trends (anemia and leukopenia), inflammatory activity, iron status, and HIV testing. Statistical tests and N(%) are not applicable to single-patient laboratory data *(single-patient results with units; reference ranges in parentheses; timepoints relative to admission. “Nadir” = lowest recorded value).*.

**Parameter**	**Findings and Course**
**Hemoglobin (g/dL)**	First documented during admission ~11–12; Nadir 6.9 (day 5, post-hematuria) and 6.45 (week 4); increased to >8.0 after transfusions [12-16].
**Hematocrit (%)**	Nadir 21.2% (concordant with Hb 6.45 g/dL); improved to >25% after transfusions [36-46].
**White blood cell count (×10^9/L)**	Developed leukopenia in week 2 (lowest ~2.5–3.0); counts recovered after discontinuation of linezolid and supportive care [4.0–11.0].
**Differential count**	Neutropenia at WBC Nadir (ANC ~1.0 ×10^9/L); lymphocyte count remained within the reference range.
**Inflammatory markers**	ESR >100 mm/h and CRP >10 mg/dL at presentation; both declined progressively with antituberculous therapy.
**Iron studies**	Pattern consistent with functional iron deficiency (elevated ferritin, low transferrin saturation) in the setting of inflammation; responded to IV ferric carboxymaltose.
**HIV serology (ELISA)**	Negative; CD4 count within normal limits.

**Abbreviations:** ANC, absolute neutrophil count; CRP, C-reactive protein; ESR, erythrocyte sedimentation rate; IV, intravenous; WBC, white blood cell.

**Table 3:** Key imaging studies and findings during admission and hospitalization (chronologic sequence relative to admission; contrast use specified where applicable). Studies are listed in strict chronological order. Findings summarize clinically relevant features that guided management (*e.g*., biopsy targets, surgery, anticoagulation). Quantitative PET metrics are presented as SUVmax without units. Imaging dates/timepoints match the narrative in the case presentation and the chronology provided in Table **1**.

**Date/Hospital Day**	**Imaging Modality**	**Key Findings**
**Admission (day 0)**	Thoracolumbar spine radiographs (AP/lateral)	Vertebroplasty cement within T12 with extension into the left T12 neural foramen; no acute fracture; old compression deformity with increased thoracic kyphosis.
**Day 1**	MRI thoracolumbar spine with contrast	Destructive spondylodiscitis at T11–T12 with disc and vertebral-body inflammation; epidural phlegmon/soft tissue causing ≈60% spinal canal narrowing, features consistent with tuberculous Pott’s disease.
**Day 4**	CT urography (abdomen/pelvis)	Bilateral hypodense renal parenchymal lesions compatible with tuberculous involvement; perinephric fat stranding; ~5-mm left proximal ureteral calculus; bladder large intraluminal clot; bilateral adrenal enlargement with right-sided calcifications and areas of hypoperfusion.
**Day 5**	Chest radiograph	Mild bilateral pleural effusions, right > left; lungs without focal consolidation (likely reactive/tuberculous effusions).
**Day 10**	Bladder ultrasound (pre-/post-void)	Echogenic mobile intravesical mass consistent with clot (~75 mL) causing incomplete emptying; marked reduction in clot volume after second cystoscopic evacuation.
**Week 3 (Day ~21)**	CT head (non-contrast)	Bilateral cortico-subcortical calcifications (right hemisphere illustrated); no acute hemorrhage or mass effect.
**Week 3**	18F-FDG PET/CT (thorax/abdomen)	Right-upper-lobe spiculated pulmonary nodule with increased uptake (SUVmax 4.0); bilateral adrenal thickening with calcifications and uptake (predominantly right, SUVmax 5.9); left thyroid nodule with increased uptake; right shoulder joint with diffuse uptake consistent with synovitis (SUVmax 7.6); several subcutaneous abdominal-wall nodules with low-grade activity.
**Week 4 (Day ~28)**	CT thoracolumbar spine (post-op)	Post-surgical instrumentation T10–L2; T12 pedicle fracture with displacement of T11/T12 screws and partial screw migration into the canal; bilateral pleural effusions with left basal atelectasis on included lung fields.
**≈3 months post-discharge**	PET/CT (thorax)	Interval response: decreased size and metabolic activity of the right-upper-lobe nodule.

**Abbreviations:** AP, anteroposterior; FDG, fluorodeoxyglucose; PET/CT, positron emission tomography/computed tomography; SUVmax, maximum standardized uptake value.

## LIST OF ABBREVIATIONS

TB: Tuberculosis
dTB: Disseminated tuberculosis
EPTB: Extrapulmonary tuberculosis
HIV: Human immunodeficiency virus
HRZE: Isoniazid rifampicin pyrazinamide ethambutol
CT: Computed tomography
MRI: Magnetic resonance imaging
PET/CT: Positron emission tomography/computed tomography
FDG: Fluorodeoxyglucose
SUVmax: Maximum standardized uptake value
US: Ultrasound
HU: Hounsfield units
ESP: Erector spinae plane
VAC: Vacuum-assisted closure
ICU: Intensive care unit
PCR: Polymerase chain reaction
NAAT: Nucleic acid amplification test
AFB: Acid-fast bacilli
ESBL: Extended-spectrum beta-lactamase
WBC: White blood cell
ANC: Absolute neutrophil count
CRP: C-reactive protein
ESR: Erythrocyte sedimentation rate
UTI: Urinary tract infection

## STANDARDS OF REPORTING

CARE guidelines were followed.
